# A Comparative Analysis of the Effects of Objective and Self-Assessed Financial Literacy on Stock Investment Return

**DOI:** 10.3389/fpsyg.2022.842277

**Published:** 2022-04-06

**Authors:** Kaicheng Liao, Yuchen Zhang, Hanyun Lei, Geng Peng, Wei Kong

**Affiliations:** ^1^School of Economics and Management, Tongji University, Shanghai, China; ^2^School of Finance, Xinjiang University of Finance and Economics, Urumqi, China; ^3^Business School, Jiangsu University of Technology, Changzhou, China; ^4^School of Economics and Management, Xi’an University of Technology, Xi’an, China

**Keywords:** composite financial literacy, objective financial literacy, risk preference, self-assessed financial literacy, stock investment return

## Abstract

Till now, comprehensive and quantitatively meaningful analyses of stock market participation outcomes of retail investors have been limited by data sources in developing countries. This article devised a special questionnaire related to stock investment to measure the financial literacy (FL) and stock investment return (SIR) for the subjects with stockownership in China and to theoretically and empirically study the effects of objective FL, self-assessed FL, and their composite FL on SIR. The results of the comparative analysis showed that self-assessed FL has a greater effect on SIR than objective FL, and the effect is mediated by risk preference. In addition, we found that competent and overconfident respondents have higher SIR, while under confident respondents cannot gain from the stock market. We also found that risk preference has a positive mediating effect in the relationship between competence and overconfidence and SIR, and a negative mediating effect in the relationship between under confidence and SIR. We thus concluded that confident investors can gain more stockholding returns *via* taking more risks regardless of the level of their actual financial knowledge. Our findings would be a meaningful complement to the studies of stock market participation.

## Introduction

There has been less research on the topic of the relationship between financial literacy (FL) and stock market participation (Van Rooij et al., 2011; Xia et al., 2014; Balloch et al., 2015; Allgood and Walstad, 2016; Zhang et al., 2021; etc.). As a classical literature about FL and stock market participation, Van Rooij et al. (2011) proved that in a Dutch household survey, those who have low FL are less likely to invest in stocks. Some studies focused on the impact of the extensions of FL on stock market participation. For example, the overconfidence of FL is positively correlated with stock market participation (Xia et al., 2014), and both the actual FL and the perceived FL appear to influence the stock market participation behavior and that the perceived FL may be as important as the actual FL (Allgood and Walstad, 2016). The general theme of these studies that appears to emerge is that FL is tightly related to stock market participation behaviors.

Furthermore, some scholars paid attention to the relationship between FL and the diversification of the portfolio. These studies found that the diversification loss of highly educated investors is actually higher than that of others (Calvet et al., 2007), and investors who claim to understand investment products can construct more efficient portfolios (Graham et al., 2009). As for whether the investors with high FL have enhanced their welfare from the stock market, the findings of von Gaudecker (2015) study indicated that households in Dutch that score high FL or rely on professionals or private contacts for advice can achieve more reasonable investment outcomes. Also, Clark et al. (2017) proved that the most financially knowledgeable investors in the United States hold 18% points more stock than their least knowledgeable counterparts and can anticipate earning 8 basis points per month more in excess returns. These studies developed countries and their stock markets. To date, only Zhang et al. (2021) studied the impact of FL on the outcomes of household stock market participation in China, which is represented by the risks of stock assets, not the realized return of stock market participation. Therefore, this article fills the void by looking into the impact of FL on stock investment return (SIR) in developing countries and paying attention to the role of risk preference in the relationship between FL and SIR. Additionally, so far no literature has discussed the relationship between the extensions of FL^1^ and SIR from the perspective of comparative analysis.

We believed that the view that individuals can benefit from the stock market should not be taken for granted, and it is important to analyze how FL is translated into stock investment outcomes. Our findings can add to the literature on the study of the relationship between FL and stock market participation behaviors. In this article, the effects of FL on SIR are theoretically analyzed according to the portfolio theory (Markowitz, 1952) and the bounded rationality theory (Simon, 1947). The results of the theoretical analysis indicate that the expected return of the portfolio is determined by FL and risk preference. Especially, objective and subjective FL can affect the expected return of the portfolio by changing the risk-taking behavior. Additionally, although many studies have proved that FL has an important impact on investment behaviors and outcomes, so far no literature has focused on the FL concerned stock investment knowledge and its extensions to comparatively discuss the relationship between FL and SIR. Considering that stockholdings have the highest proportion in the allocation of financial assets for China, we developed some items of financial knowledge related to the Chinese stock market to measure FL, which allowed us to study the different impacts of some intensions of FL on SIR. Adding this information to existing studies can substantially enhance the research on stock investment decisions. Finally, we tried to provide a new explanation toward the so-called stock-holding puzzle, i.e., the fact that many households do not hold stocks (Haliassos and Bertaut, 1995; Campbell, 2006). Therefore, our findings would be a meaningful complement to the existing literature.

## A Theoretical Analysis About the Effects of Financial Literacy on Stock Investment Return

**FIGURE 1:**
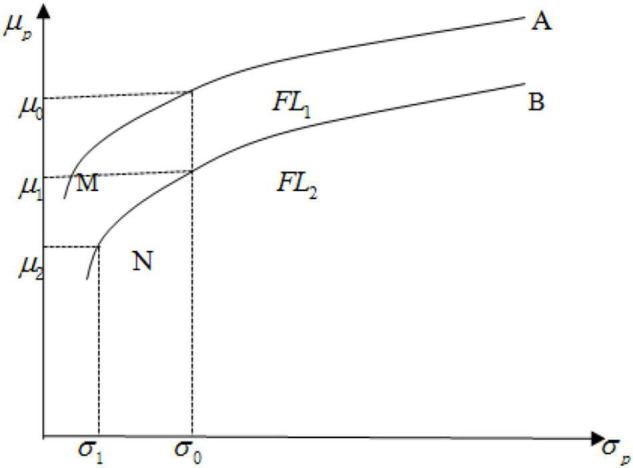
The determinants of portfolio return.

We believed that FL and risk preference are the two sources of SIR. As we know, managing a portfolio requires specific FL, in terms of basic financial knowledge and advanced investment knowledge needed to choose stocks with high quality. Low FL is likely to be associated with an inefficient portfolio, which should mean low realized investment return. In Figure 1, MA represents an efficient portfolio frontier^2^ (Markowitz, 1952), on which each point means a portfolio with a maximized return under the condition of a certain risk. But the bounded rationality theory (Simon, 1947) holds that the decision-maker pursues limited rationality, not pursues rationality to the greatest extent. The reason is that people’s cognitive ability is limited, and thus, the decision-maker can neither grasp all the information nor recognize all the knowledge and laws related to decision-making. Nevertheless, it is still reasonable to assume that the higher the level of FL, the more rational the investor’s behavior will be, and the portfolio will be more efficient. As shown in Figure 1, the portfolio frontier of an investor with *FL*_1_ (MA) is more efficient than that of an investor with *FL*_2_ (NB) because of *FL*_1_ > *FL*_2_. Compared with the portfolio on NB, those on MA are more diversified, just as proved by Calvet et al. (2007) and Graham et al. (2009). Given that the risk appetites of two investors are the same (σ_*0*_), the investor with a relatively high FL can obtain a higher expected return, i.e., μ_*0*_ > μ_*1*_. This conclusion is consistent with the evidence provided by von Gaudecker (2015). Additionally, risk preference is another important source of SIR. In Figure 1, an investor with greater risk preference (σ_*0*_) should choose a portfolio with more risk than an investor with the same FL (*FL*_2_)^3^ but with smaller risk preference (σ_*1*_). Although the portfolio is not efficient on the portfolio frontier NB, i.e., the unsystematic risks of the portfolio chosen by them are not fully diversified, the expected return μ_*1*_ might be bigger than μ_*2*_. Therefore, investors can acquire a possibly high realized SIR by taking more risks.

We believed that FL is a more important source of SIR than risk preference because it can affect the benefit of the portfolio by changing the risk-taking behavior. First of all, evidence from many empirical studies shows that risk aversion and objective FL have an inverse relation. Some experimental studies indicate that greater objective FL is associated with more patient and less risk-averse behavior (Benjamin et al., 2013; Taylor, 2016). Survey evidence also proves that FL has a positive relation with risk preference (Peng et al., 2020). Higher objective FL would then be associated with aggressive risk-taking behavior (i.e., choosing portfolios with high risk), which should make SIR higher likely. Moreover, self-assessed FL (i.e., subjective or perceived FL) might also be very useful for explaining the SIR. As we know, self-assessment of FL can be used to measure confidence. This confidence in investment knowledge is not granted to lead to poor financial decisions. Instead, Hung et al. (2009) proved that confidence in financial knowledge may improve financial decisions or outcomes because financial confidence may be needed to take an action. Similarly, individuals with high confidence level might believe that they are capable of controlling more risks, which should also induce risk-taking behaviors. Therefore, objective or self-assessed FL, which may play a more important role in stock investment, is a worthy question to be discussed.

Second, the connection of FL and stockholding return may be from the potential relation between overconfidence and risk preference. Overconfidence is usually associated with poor cognitive skills (Barber and Odean, 2001). Allgood and Walstad (2016) argued that a person with high perceived FL and low objective FL may be thought of as overconfident. Some studies on stock investment behavior and overconfidence found that overconfident individuals believe that they have better abilities to forecast future stock prices and control risks, which lead them to take riskier stock positions (Odean, 1998; Barber and Odean, 2001; Xia et al., 2014). Additionally, overconfident investors also underestimate the risk of stocks (Christelis et al., 2010), which makes them take more risks in the stock investment than under confident investors. Therefore, overconfidence may play a most important role in stock investment, and investors with overconfidence might therefore get higher realized investment return in the stock market by taking more risks.

Since objective and self-assessed FL might have different effects on the expected return of the portfolio, it is important to analyze comparatively their impact on the realized return in the stock market. In this article, we improved substantially upon the measurement of FL by considering more refined indices of FL and stock investment sophistication to analyze the impact of FL on stock investment incomes. In addition, to better understand the sources of SIR, we implemented an investigation that can provide information to assess the direction of causality between FL, its composite indices and SIR, and the mediating effect of risk preference.

## Data and Method

### Data

In this article, we included the investors in the Chinese stock market as the respondents in the survey to acquire the research data. According to the 2019 Chinese National Survey of Investors in Stock Market, there are almost 160 million investors in the stock market as of December 31, 2019, in which the proportion of individuals investing in stock directly is up to 99.76%. Thus, individuals have to assume more responsibility for their own financial wellbeing, and policymaker should attach more importance to the investor’s stockholding welfare in China. At present, there is also no public data source in China that can provide administrative data on stock portfolio information of households, similar to those used by Calvet et al. (2007), von Gaudecker (2015), and Clark et al. (2017). To rectify this lack of data, we devised a questionnaire to investigate the individual investors in the Chinese stock market. Especially, we focused on the level of investment knowledge related to stockholding to measure FL, which is more likely to affect SIR. To study the impact of FL including objective FL, subjective FL, and composite FL on stockholding return, we asked respondents with stockownership some questions about the level of objective and self-assessed financial knowledge, stockholding return, and socioeconomic characteristics. Given that risk-taking has an important association with portfolio return (Calvet et al., 2007; von Gaudecker, 2015), in this survey we also need to acquire risk attitude data to analyze the role of risk preference on the relationship between FL and stockholding return.

The questionnaire devised by us covered information about the demographic and economic characteristics of respondents and focused on FL related to stock investment, risk preference, and SIR data. The survey was divided into three stages, including pre-survey stage (from November 2019 to December 2019), field survey stage (from March 2020 to June 2020), and Internet survey stage (from March 2021 to May 2021). In the pre-survey stage, we inquired 30 experts in financial field and revised the questionnaire according to their suggestions. Then, a site investigation was made to obtain the comprehension bias and time required in the process of answering the questionnaire. In the field survey stage, feedback information of 320 respondents was used to improve the wording of each question of the questionnaire. Especially, we acquired the minimum time required to answer all the questions (10 min), which was regarded as a criterion for excluding the invalid questionnaires from the Internet survey sample. To avoid the possible impact of the COVID pandemic on the self-assessed FL and risk perception of investors, we chose a period with almost no COVID case for the third survey. The data collected with Internet surveys suffered less from reporting biases than those collected *via* telephonic interviews (Chang and Krosnick, 2009). Therefore, in the third stage, we conducted an online survey of 15,000 Chinese adults (500 per province, not including Tibet, Macau, Hong Kong, and Taiwan) who have investments in the stock market. The recruitment and random selection of participants were done by a security company cooperating with us, and then, the participants were interviewed *via* the Internet. All of the participants had Internet to invest stocks, and thus, the Internet connection rate of the participants was not considered. The regional distribution and random selection of the sample controlled the selection bias and provided it with a good representative of the population of those who actually participated in the Chinese stock market; 15,000 questionnaires were collected, and the questionnaire was judged as an invalid one if the time for answering all the questions was less than 10 min. Finally, a total of 13,911 out of 15,000 questionnaires served as a valid sample, implying a response rate of 92.74%.

### The Measurement of Financial Literacy

We developed 15 items for the measurement of objective FL by introducing and improving some questions from De Nederlandsche Bank’s Household Survey (DHS), National Financial Capability Study (NFCS), and China Household Finance Survey (CHFS). Specifically, we devised some items related to stock investment to measure whether individuals possess the necessary skills to perform well in the process of stock investment. Additionally, some questions about basic financial knowledge in DHS, NFCS, or CHFS were improved according to the Chinese context (Supplementary Appendix A1, correct answers are provided in bold, the subject of the question is provided in the front of each item, and the sources are provided in the parentheses). For example, the interest rate question was divided into three questions in our survey, including the interest rate in China (Devised by us), simple and compound interest rate calculation (Improved according to the Chinese context and similar questions in DHS, NFCS, and CHFS). In the survey, respondents chose one of the two options: (1) they do not know the answer or (2) refuse to answer. This helped respondents not choose at random. In line with previous research (Peng et al., 2018), a “1” represents a correct response and a “0” represents an incorrect response; for a “don’t know” or a “refuse to answer” response, an objective FL score was chosen and its range was [0, 15]. Responses to these questions are reported in Table 1^4^. The respondents are stock investors in our sample, which implies that they are familiar with investment knowledge about the stock market. Therefore, it is not surprising to find that most of them (93.07%) got an objective FL score above or equal to median value 8.

**TABLE 1:** The summary of the response of objective, self-assessed, and composite FL.

Objective FL score	Number	Proportion
[0−3]	51	0.37%
[4−7]	913	6.56%
[8−11]	6841	49.18%
[12−15]	6106	43.89%

**Self-assessed FL score**	**Number**	**Proportion**

1	447	3.21%
2	1156	8.31%
3	2281	16.40%
4	3203	23.02%
5	4195	30.16%
6	1954	14.05%
7	675	4.86%

**Composite FL**	**Number**	**Proportion**

Overconfidence	2141	15.39%
Under confidence	3976	28.58%
Competence	4753	34.17%
Naivety	3041	21.86%

*The observations are 13,911.*

Additionally, we focused on self-assessed FL other than objective FL, which allowed us to discern whether the ability or the confidence has a greater effect on SIR. According to the survey of NFCS, respondents in our survey were asked to assess their own financial knowledge level on a 7-point Likert item scale, whereby a “1” reflects the lowest level and a “7” reflects the highest level (Supplementary Appendix A2). It is worth mentioning that the question is located at the beginning of the FL module, before any of the questions included in the objective FL indices are asked. Thus, respondents have to assess their own knowledge before they answer the objective FL questions, which can avoid the possible impact of the objective FL questions replies on their confidence. Responses to the self-assessed FL question are reported in Table 1. The pattern of answers is much different from the objective FL. For example, only 72.09% of respondents assessed their own FL above or equal to median value 4, which is far lower than the proportion (93.07%) of the respondents with above or equal median value of objective FL. This finding indicates that many investors are not confident with their investment ability regardless of the actual level of financial knowledge.

To better understand the interaction effect between objective FL and self-assessed FL on SIR, we constructed a composite FL. Similar to the previous studies (Allgood and Walstad, 2013), the split of the sample into “Objec-high” and “Objec-low” groups was done using the mean of the objective FL to determine the sorting (Objec-high > mean and Objec-low ≤ mean). The split of the sample into “Subjec-high” and “Subjec-low” groups was based on the self-assessed FL mean (Subjec-high > mean and Subjec-low ≤ mean). Similar to Porto and Xiao (2016) study, we sorted the sample into four groups of composite FL: Objec-high and Subjec-high (defined as competence), Objec-low and Subjec-low (defined as naivety), Objec-high and Subjec-low (defined as under confidence), and Objec-low and Subjec-high (defined as overconfidence). As shown in Table 1, only 34.17% of respondents are competent in FL, and about 22% of respondents have a relatively low level of objective and self-assessed investment knowledge. Most importantly, almost 29% of respondents are under confident, which means that they might not get ideal returns in the process of stock investment.

### The Measure of Stock Investment Return

Most surveys, including DHS and NFCS, only use a yes-no test question to measure the willingness of the stock market participation of respondents. For the purpose of this research, we used a multiple-choice test question for the measurement of the realized outcome of stock market participation of the previous year, similar to CHFS 2017 (Supplementary Appendix B1). To ensure that the self-assessed outcome of realized gain/loss provided by the respondents can qualify as a precise outcome of stock investment, we asked the respondents to check the real return of the security account last year when they are investigated. Therefore, the measure of SIR of this survey is reliable to get an idea of investment ability. In addition, the greatest advantage of the SIR data allows the detailed measures to be related to the covariates the literature deems most important in the determinants of the willingness to participate in the stock market. As shown in Table 2, only 41.17% of respondents gained from the stock market, while almost 59% of respondents didn’t accumulate wealth through stockholding.

**TABLE 2:** The summary of SIR.

SIR	Number	Proportion
The loss is greater than 30%	1289	9.27%
The loss is between 20 and 30%	1031	7.41%
The loss is between 10 and 20%	1915	13.77%
The loss is between 0 and 10%	2038	14.65%
Break-even	1911	13.74%
The profit is between 0 and 10%	3077	22.12%
The profit is between 10 and 20%	1796	12.91%
The profit is between 20 and 30%	533	3.83%
The profit is greater than 30%	321	2.31%

*The observations are 13,911.*

### The Measure of Risk Preference

Like the device of the measure of risk preference in CHFS, we used a multiple-choice question with six items to ask the risk-taking willingness of respondents (refer to the first question of Supplementary Appendix B2). The statistics descriptive of the risk preference of the sample is provided in Table 3. We defined the choices of “A high-risk and high-return project” or “A slightly high-risk, slightly high-return project” as 1 or^5^ as 0, respectively. Therefore, 0 represents risk aversion, and 1 represents risk loving. In our sample, most of the respondents (70.64%) were risk averse, which was consistent with the characteristics of the population.

**TABLE 3:** The summary of risk preference.

The six items	Number	Proportion
A high-risk and high-return project	408	2.93%
A slightly high-risk, slightly high-return project	3677	26.43%
Project of average risk and average return	6529	46.93%
A slightly less risky, slightly less rewarding project	2890	20.77%
Unwilling to take any risks	312	2.24%
Don’t know	95	0.68%
**Risk preference**		
1	4085	29.36%
0	9826	70.64%

*The observations are 13,911.*

### Empirical Methods

Similar to von Gaudecker (2015) study, we argued the stock investment process in terms of a simple production. The output is a measure of investment outcome, i.e., the return of stockholding considered in Section “The measure of risk preference.” The most important independent variable in our study is FL, which is categorized into objective and self-assessed FL, and their composite indices. The other demographic independent variables identified in the literature include education, income, age, and education. A detailed definition of all variables is described in Table 4. We approximated the production function by an equation:


SIR=α+1βF1L+θR1iskpreference+ΓD1emographic

(1) $$variables+\varepsilon {}_{1}$$

**TABLE 4:** The definition of variables.

Variables	Definition
SIR	The range is [1, 9], and 1: the loss is greater than 30%, 2: the loss is between 20 and 30%, 3: the loss is between 10 and 20%, 4: the loss is between 0 and 10%, 5: break-even, 6: the profit is between 0 and 10%, 7: the profit is between 10 and 20, 8: the profit is between 20 and 30%, 9: the profit is greater than 30%.
Objective FL	The sum value of correct responses of the 15 FL questions and the range is [0, 15].
Self-assessed FL	The range is [0, 7].
Overconfidence	1: overconfidence, 0: otherwise.
Under confidence	1: under confidence, 0: otherwise.
Competence	1: competence, 0: otherwise.
Risk preference	1: ‘A high-risk and high-return project’ or ‘A slightly high-risk, slightly high-return project’, 0: otherwise.
Gender	1: male, 0: female.
Age	The range is [20, 73].
Years in stock market	2020 minus the start year of participating stock market of respondent, and the range is [0, 30].
Participation in stock market of families	1: there is any family member participating in stock market, 0: otherwise.
Registered residence	1: urban resident, 0: rural resident.
Business	1: the respondent engages in industrial or commercial production and operation projects, 0: otherwise.
Education	The educational level is measured by the learning years in school. 0: never went to school, 6: primary school, 9: junior middle school or high school graduate, 12: special school, 15: junior college, 16: college graduate, 19: post graduate degree, 23: doctor degree.
Major	1: the respondent has learning experience in economics or management major, 0: otherwise.
The understanding of economics knowledge	The range is [1, 5], and the higher value means the higher understanding of economics knowledge.
The use of economics knowledge	The range is [1, 5], and the higher value means the higher frequency of the use of economics knowledge.
Living status	The range is [1, 3], and the higher value means the better living status compared with sister/brother.
Marital status	1: Married, 0: otherwise.
Employment 1	1: The work time excesses 30 h in every week, 0: otherwise.
Employment 2	1: The work time does not excess 30 h in every week, 0: otherwise.
Health status	The range is [1, 5], and the higher value means the better status of health.
Income	1: high level of income (the choice is 6 or 7 in the household income question), 0: otherwise.
Living arrangement	1: live with spouse/partner, 0: live alone.
Children	1: there are one or more children with financial dependents, 0: otherwise.

Given that SIR is an ordered variable, and the disturbance parameter ε_1_ satisfies the normal distribution, we used the Ordered Probit model and standard maximum likelihood estimation method to estimate the regression (1).

For the test of the role of risk preference on the relationship between FL and stockholding return, we added the two regressions as follows.


(2) SIR=α+2βF2L+ΓD2emographicvariables+ε2

Riskpreference=α+3βF3L+ΓD3emographic

(3) $$variables+\varepsilon {}_{3}$$

The direct effect of FL on SIR is β_1_, the total effect of FL on SIR is β_2_, and the indirect effect of risk preference between FL and SIR is θ_1_ times β_3_. According to Baron and Kenny (1986) study, if β_2_, θ_1_, and β_3_ are statistically significant, then there is the mediating effect of risk preference between FL and SIR. Given that risk preference is a binary variable, we used the Probit model and standard maximum likelihood estimation method to estimate the regression (3).

## Results

### The Descriptive Statistics and Correlation Analysis of Variables

As shown in Table 5, the mean of the objective FL of our sample is 10.381, which implies that the level of investment knowledge of investors in the stock market is high. But, the mean of self-assessed FL of investors is not high (4.316, a little higher than the median). Especially, the average SIR of investors is lower than the median, which means low SIR of Chinese investors.

**TABLE 5:** The descriptive statistics of variables.

Variables	Observations	Mean	Std. dev.	Min	Max
SIR	13911	4.573	2.141	1	9
Objective FL	13911	10.381	2.257	2	15
Self-assessed FL	13911	4.316	1.197	1	7
Overconfidence	13911	0.172	0.301	0	1
Competence	13911	0.342	0.483	0	1
Under confidence	13911	0.276	0.415	0	1
Risk preference	13885	0.279	0.396	0	1
Gender	13911	0.609	0.441	0	1
Age	13911	33.761	9.015	20	73
Years in stock market	13911	9.143	7.109	0	30
Participation in stock market of families	13911	0.598	0.436	0	1
Registered residence	13911	0.831	0.371	0	1
Business	13911	0.129	0.315	0	1
Education	13857	16.736	2.348	3	23
Major	13911	0.453	0.474	0	1
The understanding of economics knowledge	13812	3.517	0.691	1	5
The use of economics knowledge	13885	3.013	0.756	1	5
Living status	12841	2.517	0.554	1	3
Marital status	13839	0.778	0.437	0	1
Employment 1	13885	0.781	0.389	0	1
Employment 2	13885	0.149	0.337	0	1
Health status	13885	3.804	0.761	1	5
Income	12897	0.459	0.437	0	1
Living arrangement	13911	0.672	0.458	0	1
Children	13812	0.658	0.439	0	1

For simplicity, the results of the Pearson’s correlation analysis of some core variables are reported in Table 6. The relationship between objective and self-assessed FL and SIR is significantly positively correlated, but the coefficient of objective FL is less than the self-assessed FL. Also, the significantly negative coefficient between under confidence and SIR implies that under confident investors cannot gain from the stock market. In addition, the correlation coefficient of objective FL and risk preference is insignificant, while the coefficient of self-assessed FL and risk preference is significantly positive. This finding indicates that the investors with high self-assessed FL are more risk loving. Similarly, the overconfident and competent investors show higher risk preference than under confident investors. These results imply that risk preference might play an important role in the relationship between FL and SIR.

**TABLE 6:** The correlation analysis of some core variables.

	SIR	Self-assessed FL	Objective FL	Overconfidence	Competence	Underconfidence	Risk preference
SIR	1						
Self-assessed FL	0.095***	1					
Objective FL	0.085**	0.320***	1				
Overconfidence	0.015**	0.274***	−0.436***	1			
Competence	0.038**	0.507***	0.454***	−0.514***	1		
Underconfidence	−0.027*	−0.434***	0.186***	−0.210***	−0.388***	1	
Risk preference	0.106***	0.186***	0.069	0.110***	0.097***	−0.109***	1

*, **, and *** indicate significance at the 10, 5, and 1% levels, respectively.

### Objective and Self-Assessed Financial Literacy and Stock Investment Return

As indicated by the findings from Gallery et al. (2011), only 41% of those respondents with a good or very good self-assessed FL had scores on the specific investment questions in the highest two quintiles. Van Rooij et al. (2011) and Lusardi and Mitchell (2017) found that there is a positive relationship between subjective and objective FL, but the cross-tabulations of scores show considerable percentages of respondents in each possible combination. The relationship between the subjective and objective measures of FL in our study is also positive, but the correlation coefficient is small^6^. Therefore, self-assessed FL is not simply another measure of actual FL. The results in Table 7 show that objective FL and self-assessed FL have positive relationships with SIR, but the impact of objective FL on SIR is significantly lower than the self-assessed FL^7^. We thus concluded that the SIR resulting from objective FL is much less than that from self-assessed FL. This finding means that confidence has a great impact on the investment return in the stock market.

**TABLE 7:** The Ordered Probit regression results of the relationship between self-assessed and objective FL and SIR.

Independent variables	The dependent variable is SIR					
	(1)		(2)		(3)	
Self-assessed FL (a)	0.198***	(0.032)			0.183***	(0.037)
Objective FL (b)			0.099***	(0.017)	0.077***	(0.011)
a–b					0.106***	[0.000]
Risk preference	0.251***	(0.054)	0.255***	(0.054)	0.251***	(0.054)
Gender	0.131**	(0.052)	0.134**	(0.052)	0.129**	(0.052)
Age	−0.017	(0.015)	−0.017	(0.015)	−0.017	(0.015)
Years in stock market	0.014	(0.016)	0.013	(0.016)	0.014	(0.016)
Participation in stock market of families	0.117**	(0.051)	0.115**	(0.051)	0.115**	(0.052)
Registered residence	0.127	(0.167)	0.126	(0.167)	0.124	(0.167)
Business	0.115	(0.104)	0.124	(0.102)	0.122	(0.107)
Education	0.113**	(0.048)	0.112***	(0.041)	0.112***	(0.041)
Major	−0.203**	(0.088)	−0.208**	(0.086)	−0.196**	(0.085)
The understanding of economics knowledge	0.154**	(0.062)	0.164***	(0.063)	0.161***	(0.062)
The use of economics knowledge	0.203***	(0.063)	0.186***	(0.062)	0.217***	(0.063)
Living status	−0.061	(0.069)	−0.065	(0.061)	−0.063	(0.071)
Marital status	−0.273***	(0.101)	−0.276***	(0.102)	−0.276***	(0.102)
Employment 1	−0.541***	(0.106)	−0.456***	(0.106)	−0.470***	(0.106)
Employment 2	−0.193	(0.119)	−0.179	(0.121)	−0.174	(0.121)
Health status	0.051	(0.086)	0.053	(0.085)	0.051	(0.087)
Income	−0.087	(0.053)	−0.100*	(0.053)	−0.083	(0.053)
Living arrangement	0.175**	(0.080)	0.172**	(0.080)	0.172**	(0.080)
Children	0.169**	(0.079)	0.174**	(0.080)	0.176**	(0.080)
Observations	12841		12841		12841	
Pseudo *R*^2^	0.066		0.065		0.067	
Wald Chi^2^	73.74***	[0.000]	73.42***	[0.000]	83.43***	[0.000]
VIF	1.80		1.62		1.78	

The heteroskedasticity robust standard errors are reported in parentheses, the numbers in the square bracket are p-value of the F-test. *, **, and *** indicate significance at the 10, 5, and 1% levels, respectively. The variance inflation factor (VIF) indicates that there is no multicollinearity in every regression.

### The Mediating Effect of Risk Preference Between Objective and Self-Assessed Financial Literacy and Stock Investment Return

Note that risk attitude is always an important predictor of SIR; those who display higher risk loving are more likely to obtain higher return in stock investment. As discussed in the “Introduction” section, both objective FL and self-assessed FL can bring stock returns by making portfolios with more risks, and thus, risk preference might be a mediator between objective and self-assessed FL and SIR. But the Panel A in Table 8 shows that there is no relationship between objective FL and risk preference, which means that individuals with high objective FL do not gain from the stock market by taking more risks. Given the positive relationship between objective FL and SIR, this finding implies that individuals with high objective FL can only accumulate wealth from stock investment by constructing an effective portfolio than by making a portfolio with more risks. Nevertheless, individuals with high self-assessed FL can get excess returns by taking more risks. The proportion of mediating effect in Panel B of Table 8 shows that 25.28% of returns are from risk taking in the relationship between investors’ confidence and SIR.

**TABLE 8:** The mediating effect of risk preference in the relationship between FL and SIR.

Panel A: The dependent variable is risk preference				
**Independent variables**	(1)		(2)	
Self-assessed FL	0.267***	(0.091)		
Objective FL			0.034	(0.102)
Gender	0.188***	(0.048)	0.201***	(0.047)
Age	−0.015	(0.013)	−0.015	(0.014)
Years in stock market	0.052	(0.053)	0.058	(0.053)
Participation in stock market of families	−0.136	(0.148)	−0.137	(0.148)
Registered residence	0.021**	(0.010)	0.065***	(0.011)
Business	−0.094	(0.063)	−0.061	(0.064)
Education	−0.096	(0.129)	−0.093	(0.131)
Major	0.125	(0.154)	0.126	(0.154)
The understanding of economics knowledge	0.152***	(0.055)	0.161***	(0.057)
The use of economics knowledge	0.063	(0.058)	0.077	(0.057)
Living status	0.043	(0.055)	0.047	(0.056)
Marital status	−0.033	(0.093)	−0.032	(0.094)
Employment 1	0.114	(0.097)	0.165*	(0.098)
Employment 2	−0.130	(0.109)	−0.133	(0.112)
Health status	−0.051	(0.047)	−0.050	(0.047)
Income	0.152***	(0.048)	0.150***	(0.049)
Living arrangement	−0.023	(0.073)	0.013	(0.074)
Children	0.137	(0.174)	0.133	(0.174)
Constant	2.202*	(1.308)	3.657***	(1.305)
Observations	12841		12841	
Pseudo *R*^2^	0.1020		0.0931	
Wald Chi^2^	53.72***	[0.000]	49.04***	[0.000]
VIF	1.82		1.83	

**Panel B: The test results of mediating effect**				

**Y = SIR**	**Indirect effect**	**Direct effect**	**Total effect**	**Proportion of mediating effect**

M = risk preference	X → M → Y	X + M → Y	X → Y	
X = self-assessed FL	0.067** (0.027)	0.198*** (0.032)	0.265*** (0.016)	25.28%

The heteroskedasticity robust standard errors are reported in parentheses, the numbers in the square bracket are p-value of the F-test. *, **, and *** indicate significance at the 10, 5, and 1% levels, respectively. Because the risk preference is a dummy variable, the results of Panel A are obtained by Probit regression. In addition, the results of Panel B are obtained by mediating effect regression. The variance inflation factor (VIF) indicates that there is no multicollinearity in every regression.

Calvet et al. (2007) found that the diversification loss of highly educated investors is actually higher than that of others because they are more exposed to risk but reach higher returns. They use education as the proxy of FL, which is regarded as the main reason for why our findings are not consistent with theirs. But von Gaudecker (2015) provided the results that many Dutch households reach reasonably effective investment outcomes in terms of the risk-return trade-off by choosing a very low level of risk, and others by turning to external help. He argued that both strategies are consistent with a rational response to poor self-perceived investment skills. Our results are similar to such explanation, i.e., respondents with low self-assessed FL always choose a portfolio with low risk.

### Composite Financial Literacy and Stock Investment Return

As shown in Table 9, overconfidence and competence have a significantly positive impact on SIR, while underconfidence has no influence on it. In addition, although the impact of overconfidence on SIR is greater than competence, the difference is not significant. The comparison of the coefficients of the three composite FL measures is important because it proves that the better outcomes are concentrated among those who have a high level of self-assessed FL regardless of their actual ability as applied to stock investment matters^8^.

**TABLE 9:** The Ordered Probit regression results of the relationship between composite FL and SIR.

	The dependent variable is SIR							
Independent variables	(1)		(2)		(3)		(4)	
Overconfidence (c)	0.254***	(0.058)					0.195**	(0.083)
Underconfidence (d)			−0.109	(0.079)			−0.138	(0.104)
Competence (e)					0.226***	(0.051)	0.174**	(0.075)
c–e							0.021	[0.833]
Risk preference	0.358***	(0.054)	0.368***	(0.054)	0.352***	(0.053)	0.347***	(0.055)
Gender	0.139***	(0.045)	0.136**	(0.052)	0.137**	(0.052)	0.136**	(0.052)
Age	−0.017	(0.014)	−0.017	(0.015)	−0.017	(0.015)	−0.018	(0.015)
Years in stock market	0.014	(0.016)	0.014	(0.015)	0.013	(0.016)	0.014	(0.016)
Participation in stock market of families	0.117**	(0.052)	0.118**	(0.051)	0.117**	(0.052)	0.118**	(0.052)
Registered residence	0.131	(0.167)	0.133	(0.167)	0.131	(0.167)	0.133	(0.167)
Business	0.115	(0.168)	0.115	(0.168)	0.116	(0.168)	0.116	(0.168)
Education	0.112***	(0.041)	0.113***	(0.041)	0112***	(0.042)	0.112***	(0.041)
Major	−0.189**	(0.089)	−0.189**	(0.087)	−0.187**	(0.086)	−0.188**	(0.089)
The understanding of economics knowledge	0.179***	(0.062)	0.167***	(0.062)	0.165**	(0.063)	0.165**	(0.063)
The use of economics knowledge	0.129**	(0.063)	0.125**	(0.063)	0.128**	(0.062)	0.125**	(0.063)
Living status	0.086	(0.060)	0.087	(0.060)	0.087	(0.060)	0.086	(0.061)
Marital status	−0.272***	(0.101)	−0.271***	(0.102)	−0.273***	(0.102)	−0.272***	(0.102)
Employment 1	−0.547***	(0.106)	−0.571***	(0.106)	−0.536***	(0.106)	−0.560***	(0.106)
Employment 2	−0.124	(0.119)	−0.128	(0.120)	−0.121	(0.119)	−0.126	(0.120)
Health status	0.019	(0.051)	0.019	(0.051)	0.020	(0.051)	0.020	(0.051)
Income	−0.011	(0.053)	−0.010	(0.054)	−0.010	(0.053)	−0.010	(0.053)
Living arrangement	0.177**	(0.080)	0.181**	(0.080)	0.179**	(0.080)	0.181**	(0.080)
Children	0.165**	(0.080)	0.166**	(0.079)	0.168**	(0.080)	0.167**	(0.080)
Observations	12841		12841		12841		12841	
Pseudo *R*^2^	0.0632		0.0639		0.0636		0.0640	
Wald Chi^2^	76.23***	[0.000]	69.63***	[0.000]	65.30***	[0.000]	66.75***	[0.000]
VIF	1.80		1.79		1.80		1.89	

The heteroskedasticity robust standard errors are reported in parentheses, the numbers in the square bracket are p-value of the F-test. ** and *** indicate significance at the 5 and 1% levels, respectively. The variance inflation factor (VIF) indicates that there is no multicollinearity in every regression.

Similarly, we discussed the mediating effect of risk preference between composite FL and SIR. Panel A of Table 10 indicates that overconfidence and competence have a positive relationship with risk preference, while underconfidence has a negative relationship with it. The test results of mediating effect in Panel B of Table 10 further show that there is a negative mediating effect of risk preference in the relationship between underconfidence and SIR. In addition, the mediating effect of risk preference in the relationship between competence and SIR (42.34%) is smaller than the effect in the relationship between overconfidence and SIR (43.05%). Given that investors with high self-assessed FL are more risk loving, it is not surprising that risk preference has a greater mediating effect for overconfidence than for competence.

**TABLE 10:** The mediating effect of risk preference in the relationship between composite FL and SIR.

Panel A: The dependent variable is risk preference						
**Independent variables**	**(1)**		**(2)**		**(3)**	
Overconfidence	0.535***	(0.101)				
Underconfidence			−0.376***	(0.065)		
Competence					0.473***	(0.119)
Gender	0.196***	(0.047)	0.201***	(0.048)	0.197***	(0.048)
Age	−0.014	(0.014)	−0.015	(0.014)	−0.015	(0.014)
Years in stock market	0.054	(0.053)	0.054	(0.053)	0.058	(0.053)
Participation in stock market of families	−0.132	(0.148)	−0.136	(0.148)	−0.137	(0.148)
Registered residence	0.061***	(0.009)	0.062***	(0.010)	0.067***	(0.011)
Business	−0.097	(0.063)	−0.094	(0.063)	−0.081	(0.063)
Education	−0.077	(0.130)	−0.091	(0.130)	−0.097	(0.132)
Major	0.127	(0.154)	0.124	(0.154)	0.127	(0.154)
The understanding of economics knowledge	0.175***	(0.057)	0.161***	(0.057)	0.158***	(0.058)
The use of economics knowledge	0.067	(0.057)	0.074	(0.057)	0.077	(0.057)
Living status	0.042	(0.056)	0.050	(0.056)	0.048	(0.056)
Marital status	−0.027	(0.093)	−0.030	(0.094)	−0.031	(0.094)
Employment 1	0.139	(0.098)	0.112	(0.097)	0.143	(0.099)
Employment 2	−0.151	(0.110)	−0.142	(0.111)	−0.136	(0.111)
Health status	−0.058	(0.047)	−0.051	(0.047)	−0.050	(0.047)
Income	0.149***	(0.048)	0.150***	(0.049)	0.150***	(0.049)
Living arrangement	0.034	(0.073)	0.058	(0.074)	0.045	(0.073)
Children	0.120	(0.173)	0.131	(0.173)	0.133	(0.173)
Constant	0.348	(0.286)	0.413	(0.286)	0.410	(0.286)
Observations	12841		12841		12841	
Pseudo *R*^2^	0.0997		0.0935		0.0933	
Wald Chi^2^	52.50***	[0.000]	49.26***	[0.000]	49.13***	[0.000]
VIF	1.82		1.82		1.83	

**Panel B: The test results of mediating effect**						

Y = SIR		**Indirect effect**		**Direct effect**	**Total effect**	**Proportion of mediating effect**
M = risk preference		X → M → Y		X + M → Y	X → Y	
X = overconfidence		0.192*** (0.046)		0.254*** (0.058)	0.446*** (0.058)	43.05%
X = underconfidence		−0.138*** (0.031)		−0.109 (0.079)	−0.247*** (0.080)	55.87%
X = competence		0.166*** (0.049)		0.226*** (0.051)	0.392*** (0.051)	42.34%

The heteroskedasticity robust standard errors are reported in parentheses, the numbers in the square bracket are p-value of the F-test. *** indicate significance at the 1% level. Because the risk preference is a dummy variable, the results of Panel A are obtained by Probit regression. In addition, the results of Panel B are obtained by mediating effect regression. The variance inflation factor (VIF) indicates that there is no multicollinearity in every regression.

### Robustness Checks

#### Endogeneity Analysis

As discussed by Van Rooij et al. (2011), literacy is not an exogenous characteristic because literacy can itself be affected by financial behavior. As for our data, individuals with high SIR might mean they have rich experience in stock investment, and they can learn *via* experience to increase the level of investment knowledge. To solve this problem, we collected additional information that served as an instrument variable for FL.

To be able to rely on the measures of FL that are exogenous with respect to SIR, we asked respondents about the learning experience in finance or economics of their parents. Specifically, we collected information on whether the parents have ever studied in the major of finance or economics. We used this information as an instrument variable for the objective FL of the respondent. The experience of his or her parents is not under the control of the respondent and is thus exogenous with respect to the respondent’s action, but the respondent can learn from his or her parents, thus increasing his or her own FL. In order to ensure robust results, we used both 2sls and GMM to perform the endogeneity test. For simplicity, we reported only the estimates of the new controls and the regressions.

The first-stage regressions of the two methods reported in Table 11 show that not only our instrument is statistically significant but the *F*-statistics are high and above or in range with the value recommended to avoid the problem of weak instruments (Bound et al., 1995; Staiger and Stock, 1997). The estimates in the second stage show that the relationship between objective FL and SIR remains positive and statistically significant in the two regressions. Moreover, the exogeneity test is not rejected. The results of the Hansen *J*-test show that the over-identifying restrictions are also not rejected. Therefore, the objective FL is not an endogenous variable in the empirical analysis, and all the regression results above are credible.

**TABLE 11:** The comparison of 2sls and GMM estimation.

Variables	First stage		Second stage		First stage		Second stage	
	OLS(1)		OLS(2)		GMM(1)		GMM(2)	
Objective FL			0.121***	(0.016)			0.111***	(0.017)
Parents’ experience	1.661***	(0.528)			1.660**	(0.829)		
Control variables	Yes		Yes		Yes		Yes	
Constant	10.248***	(1.375)	0.663	(0.714)	10.248***	(1.510)	0.640	(0.789)
Observations	12841	12841	12841	12841
*R* ^2^	0.1466	0.1471	0.1466	0.1530
*F*	16.42***	[0.000]			18.63***	[0.000]		
Wald chi^2^			37.26***	[0.000]			44.08***	[0.000]
Hanse *J*-test *p*-value			[0.478]			[0.358]
*P*-value of exogeneity test			[0.564]			[0.771]

The dependent variable of the first stage is objective FL, and the dependent variable of the second stage is SIR. In the regression of the second stage, parents’ experience is used as the instrument variable of objective FL. The heteroskedasticity robust standard errors are reported in parentheses, the numbers in the square bracket are p-value of the F-test. ** and *** indicate significance at the 5 and 1% levels, respectively.

#### The Different Measures of Core Variables

Stock investment return, composite FL, and risk preference are core variables in this study, and they can be measured by other methods. For SIR, we used a dummy variable other than an ordered variable to measure it. Specifically, the choices for the items with positive return (including break-even) in the SIR question are defined as 1 and others are defined as 0. Then, we used the Probit model to estimate the regressions with SIR as the dependent variable.

The method used to measure composite FL in this article was developed by Allgood and Walstad (2013). Allgood and Walstad (2013) used a sample with 28,146 adults to calculate the average of FL, and then, they used it as the benchmark to split the sample into high or low group. But our sample has only 13,911 observations, so the average FL of the sample with relatively small scale may not have the representative. We thus used the median value 8 as the benchmark to define the low or high level for objective FL and the median value 4 for self-assessed FL, and then, the four new groups of composite FL were obtained.

For risk preference, we used another question to investigate the risk attitude of respondents because the question of risk preference described in Section “The Measurement of Financial Literacy” is crude. The new question is described as the second question in Supplementary Appendix B2. We coded the first choice as 4, the second as 3, the third as 2, the fourth as 1, and the fifth as 0, and then, we obtained an ordered data of risk preference. Obviously, the respondent with a bigger value is more risk loving.

Using the new measures of SIR, composite FL, and risk preference, we conducted all the regressions again. Also, we found that the same pattern holds for the different measures, which proved that our results are valid and robust^9^.

## Conclusion and Discussion

In this study, the theoretical analysis shows that FL and risk preference are the two sources of SIR, but FL can affect SIR by influencing risk tolerance. This finding implies that FL might play a more important role on SIR than risk preference. Then, the detailed stock investor’s information is obtained through a survey, which allows us to examine the relation between FL and SIR of stock investors in China. With our data set, FL can be categorized into objective and self-assessed FL and four groups of composite FL. Especially, we discerned that the high SIR is due to that investors are better equipped to make their own investment decisions or they are more confident to purchase risky stocks. If the former case is proved, financial education programs could be of help (Tang et al., 2010). But if the investor’s confidence is the key, the policies would target the enhancement of the investment confidence.

The findings show that objective FL has a positive impact but with a low significant level (10%) on stockholding return, which is consistent with the results of von Gaudecker (2015) and Clark et al. (2017). Most importantly, we found that the self-assessed FL has a more strong impact with high significant level (1%) on SIR and risk preference has a significant mediating effect in the relationship between them. The results show that the correlation between self-assessed FL and stockholding return is mainly driven by risk-taking willingness. Since the objective FL can improve SIR, the findings about the mediating effect of risk preference imply that stockholding return is determined by both risk-taking willingness and investment ability. In addition, we found that overconfident and competent investors can get a higher return by stockholding, while underconfident investors cannot accumulate wealth from the stock market. Also, we found that risk preference has a significant mediating effect in the relationship between composite FL and SIR. The robustness tests show that these results are robust for the different measures of SIR, composite FL, and risk preference.

Despite SIR in this study is a coarse indicator for the measurement of the outcome of stock market participation, we obtained some meaningful conclusions. The findings showed that the effect of self-assessed FL on SIR is larger than the objective FL. In addition, our results showed that SIR is also determined by the combination of actual literacy and confidence. Most importantly, when considering other measures of composite FL, SIR, and risk preference, the same basic patterns emerge. A plausible interpretation of these patterns is the impact of the self-assessed FL, and the combination of actual and self-assessed FL on the willingness to taking risks, just as the illustrations of theoretical analysis. Investors with high level of self-assessed financial knowledge trust their own capabilities to control more risks, which may incur higher returns. Consistent with this interpretation, the mediating effect of risk preference is proved to be economically and statistically significant in the relationship between self-assessed FL, composite FL, and SIR. The pattern also suggests that high SIR most often should reflect more risk of the portfolio as opposed to optimal strategies. That the investment confidence turns out to be much more important means that increasing it would do much for portfolio outcomes, regardless of the level of actual FL of investors. Additionally, our results show that individuals might shy away from the stock market because the ones with low self-confidence level cannot get an ideal return by stockholding.

Our findings have a particular policy implication. The results of this study show that confidence could bring a higher realized return from stock market investment than the actual level of financial knowledge. So policies that aim to enhance the confidence investors could substantially improve the welfare of stock market participation in China. Therefore, we suggest that both FL and confidence—be it from perfect security market supervision, public information disclosure, etc.—are potential starting points for policies seeking to enhance households welfare from the stock market in China.

We plan to expand this work in several directions. First, we will examine the relationship between FL and specific portfolio and explore whether difficulties in low financial sophistication related to stock investment affect the ability to construct an efficient portfolio. Moreover, we will assess whether overconfidence or underconfidence has an effect on the efficient portfolio construction.

## Data Availability Statement

The raw data supporting the conclusions of this article will be made available by the authors, without undue reservation, to any qualified researcher.

## Author Contributions

KL: acquisition of original data, software, formal analysis, methodology, and manuscript revision. YZ: reference management and manuscript revision critically. GP: conceptualization, writing – review and editing, methodology, funding acquisition, and manuscript revision. HL and WK: data calculation and writing. All authors contributed to the article and approved the submitted version.

## Conflict of Interest

The authors declare that the research was conducted in the absence of any commercial or financial relationships that could be construed as a potential conflict of interest.

## Publisher’s Note

All claims expressed in this article are solely those of the authors and do not necessarily represent those of their affiliated organizations, or those of the publisher, the editors and the reviewers. Any product that may be evaluated in this article, or claim that may be made by its manufacturer, is not guaranteed or endorsed by the publisher.

## Footnotes

1 In this study, the extensions of FL consist of self-assessed FL, overconfidence, underconfidence, competence, and naivety.

2 Portfolio theory initiated by Markowitz in 1952 aims to construct an efficient portfolio by selecting a package of securities with a specific investment proportion.

3 This implies that they confront the same portfolio frontier, i.e., NB in Figure 1.

4 We calculated Cronbach’s alpha for the answer results of these 15 questions. The Cronbach’s alpha is 0.832, which imply that there is a strong reliability and internal consistency for the measurement of objective FL.

5 The respondents answering “don’t know” are excluded from the sample.

6 As shown in Table 6, the correlation coefficient between the self-assessed and objective FL is only 0.32.

7 The difference between the coefficients of self-assessed FL and objective FL is 0.106, which is statistically significant.

8 According to the definition of the four composite FL, overconfident and competent investors have relatively high self-assessed FL level.

9 For brevity, we do not report the robustness check results in this study, but they can be provided upon request.
