# Supplementary material for: A Comparative Analysis of the Effects of Objective and Self-Assessed Financial Literacy on Stock Investment Return
Source: Front Psychol. 2022 Apr 6;13:842277. doi: 10.3389/fpsyg.2022.842277 (PMC9019082; doi:10.3389/fpsyg.2022.842277)
Supplement: Supplementary file 1 [file Presentation_1.pdf]

Appendix A. Constructing indices for financial literacy

A.1. Objective financial literacy

1. Interest rate in China: What do you think is the interest rate of one-year fixed deposit? (Devised by us)

[ ] Less than 1% [ ] **1%-3%** [ ] 3%-6% [ ] 6%-9%

[ ] More than 9% [ ] Don't know [ ] Refuse to answer

2. Simple interest rate calculation: Suppose you have a one-year fixed deposit of 10000 Yuan, and the annual interest rate is 3%. If you don't withdraw in advance, how much money will you have when the deposit matures? (Improved according to Chinese context and the similar question in DHS, NFCS and CHFS)

[ ] Less than 10300 Yuan [ ] **10300 Yuan** [ ] More than 10300 Yuan

[ ] Don't know [ ] Refuse to answer

3. Compound interest rate calculation: After the above deposit matures, if you deposit the money in the account with a fixed term of 2 years and the annual interest rate is still 3%, how much is the total principal and interest in the account after 2 years? (Improved according to Chinese context and the similar question in DHS)

[ ] Less than 10900 Yuan [ ] 10900 Yuan [ ] **More than 10900 Yuan**

[ ] Don't know [ ] Refuse to answer

4. Inflation: If the annual interest rate of your bank deposit account is 3% and the inflation rate is 5% per year, what can you buy with the money in this account after one year compared with that of one year ago? (Improved according to Chinese context and the similar question in DHS, NFCS and CHFS)

[ ] More than a year ago [ ] Equal to a year ago [ ] **Less than a year ago**

[ ] Don't know [ ] Refuse to answer

5. Money illusion: Suppose that in the year 2010, your income has doubled and prices of all goods have doubled too. In2010, how much will you be able to buy with your income? (Improved according to Chinese context and the similar question in DHS)

[ ] More than today [ ] **Equal to today** [ ] Less than today

[ ] Don't know [ ] Refuse to answer

6. Time value of money: Suppose Zhang inherit 100000 Yuan now, and Li will inherit 100000 Yuan after three years. Whose value of inheritance is higher? (Improved according to Chinese context and the similar question in DHS)

[ ] **Zhang's** [ ] Li's [ ] They inherit the same value

[ ] Don't know [ ] Refuse to answer

7. Relation between risk and return: In general, investments that are riskier tend to provide higher returns over time than investments with less risk. (Devised by us)

[ ] **True** [ ] False [ ] Don’t know [ ] Refuse to answer

8. Simpler comparison of risk: Is this statement True or False? Buying a single company’s stock usually provides a safer return than a stock mutual fund. (Improved according to Chinese context and the similar question in DHS)

[ ] True [ ] **False** [ ] Don’t know [ ] Refuse to answer

9. More complex comparison of risk: In general, which of the following assets is the most risky? (Improved according to Chinese context and the similar question in DHS)

[ ] Deposit [ ] Bond [ ] **Stock** [ ] Fund [ ] Don’t know [ ] Refuse to answer

10. Function of bank: Which of the following banks has the function of formulating and implementing monetary policy? (Devised by us)

[ ] Bank of China [ ] Industrial and Commercial Bank of China

[ ] **People's Bank of China** [ ] China Construction Bank

[ ] None of them [ ] Don’t know [ ] Refuse to answer

11. The nature of stock: If you buy a company’s stock (Improved according to Chinese context and the similar question in DHS)

[ ] You have lent money to the company

[ ] **You have been a shareholder of the company**

[ ] For a long time, you have been a shareholder, while for a short term, you have lent money to the company

[ ] Don’t know

[ ] Refuse to answer

12. Fund: What is the correct description of fund? (Devised by us)

[ ] The future performance of fund with low price will be high

[ ] **In general, a fund can invest in several assets**

[ ] The fund is able to provide a breakeven rate of return based on past performance

[ ] None of the above is true

[ ] Don’t know

[ ] Refuse to answer

13. Discount: Suppose we want to get 10000 Yuan currency in the later five years. If the annual interest rate is 6%, the current principal is (Devised by us)

[ ] Less than 7000 Yuan [ ] 7000 Yuan [ ] **More than 7000 Yuan**

[ ] Don’t know [ ] Refuse to answer

14. The return of financial product: Which of the following description of financial products is correct? (Devised by us)

[ ] **The financial products of bank may lose money**

[ ] At least it won't lose money

[ ] The expected rate of return is the actual rate of return

[ ] Don’t know

[ ] Refuse to answer

15. Bond: How do bond prices usually change if interest rates rise? (Improved according to Chinese context and the similar question in DHS and NFCS)

[ ] Rise [ ] **Fall** [ ] Stay the same

[ ] There is no relationship between them [ ] Don’t know [ ] Refuse to answer

A.2. Self-assessed financial literacy

On a scale from 1 to 7, where 1 means very low and 7 means very high, how would you assess your overall knowledge level about stock investment?

Very low Very high

[ ] 1 [ ] 2 [ ] 3 [ ] 4 [ ] 5 [ ] 6 [ ] 7

[ ] Don’t know [ ] Refuse to answer

Appendix B. Survey questions of some core variables

B.1. Stock investment return

Last year, which range is your realized return from Chinese security market within?

[ ] The loss is greater than 30%

[ ] The loss is between 20%-30%

[ ] The loss is between 10%-20%

[ ] The loss is between 0-10%

[ ] Break-even

[ ] The profit is between 0-10%

[ ] The profit is between 10%-20%

B.2. Risk preference

1. If you have a sum of money for investment, which project would you most like to choose?

[ ] A high-risk and high-return project

[ ] A slightly high-risk, slightly high-return project

[ ] Project of average risk and average return

[ ] A slightly less risky, slightly less rewarding project

[ ] Unwilling to take any risks

[ ] Don’t know

2. There is a game, in which you have a 50% chance to get a loss with 100 Yuan and a 50% chance to get a gain with X Yuan. How much do you want X to be at least in order to participate in this game?

[ ] 150 Yuan [ ] 200 Yuan [ ] 250 Yuan [ ] 300 Yuan [ ] more than 300 Yuan

B.3. Parent’s learning experience

Have your parents ever studied in the major of finance or economics?

[ ] Yes [ ] No
